# Historical Biogeography and Diversification of Truffles in the *Tuberaceae* and Their Newly Identified Southern Hemisphere Sister Lineage

**DOI:** 10.1371/journal.pone.0052765

**Published:** 2013-01-02

**Authors:** Gregory Bonito, Matthew E. Smith, Michael Nowak, Rosanne A. Healy, Gonzalo Guevara, Efren Cázares, Akihiko Kinoshita, Eduardo R. Nouhra, Laura S. Domínguez, Leho Tedersoo, Claude Murat, Yun Wang, Baldomero Arroyo Moreno, Donald H. Pfister, Kazuhide Nara, Alessandra Zambonelli, James M. Trappe, Rytas Vilgalys

**Affiliations:** 1 Deparment of Biology, Duke University, Durham, North Carolina, United States of America; 2 University of Minnesota, Department of Plant Biology, St. Paul, Minnesota, United States of America; 3 Instituto Tecnológico de Ciudad Victoria, Tamaulipas, México; 4 Department of Forest Ecosystems and Society, Oregon State University, Corvallis, Oregon, United States of America; 5 Instituto Multidisciplinario de Biología Vegetal, Córdoba, Argentina; 6 Institute of Ecology and Earth Sciences and the Natural History Museum of Tartu University, Tartu, Estonia; 7 Institute National de la Recherche Agronomique et Nancy University, Champenoux, France; 8 New Zealand Institute for Plant & Food Research Ltd, Christchurch, New Zealand; 9 Department of Plant Biology, University of Córdoba, Córdoba, Spain; 10 Farlow Herbarium, Harvard University, Cambridge, Massachusetts, United States of America; 11 Department of Natural Environmental Studies, Graduate School of Frontier Science, The University of Tokyo, Chiba, Japan; 12 Dipartimento di Science Agrarie, Università di Bologna, Bologna, Italy; 13 Institute of Systematic Botany, University of Zürich, Zürich, Switzerland; 14 Department of Plant Pathology, University of Florida, Gainesville, Florida, United States of America; University of California Riverside, United States of America

## Abstract

Truffles have evolved from epigeous (aboveground) ancestors in nearly every major lineage of fleshy fungi. Because accelerated rates of morphological evolution accompany the transition to the truffle form, closely related epigeous ancestors remain unknown for most truffle lineages. This is the case for the quintessential truffle genus *Tuber*, which includes species with socio-economic importance and esteemed culinary attributes. Ecologically, *Tuber* spp. form obligate mycorrhizal symbioses with diverse species of plant hosts including pines, oaks, poplars, orchids, and commercially important trees such as hazelnut and pecan. Unfortunately, limited geographic sampling and inconclusive phylogenetic relationships have obscured our understanding of their origin, biogeography, and diversification. To address this problem, we present a global sampling of *Tuberaceae* based on DNA sequence data from four loci for phylogenetic inference and molecular dating. Our well-resolved *Tuberaceae* phylogeny shows high levels of regional and continental endemism. We also identify a previously unknown epigeous member of the *Tuberaceae* – the South American cup-fungus *Nothojafnea thaxteri* (E.K. Cash) Gamundí. Phylogenetic resolution was further improved through the inclusion of a previously unrecognized Southern hemisphere sister group of the *Tuberaceae*. This morphologically diverse assemblage of species includes truffle (e.g. *Gymnohydnotrya* spp.) and non-truffle forms that are endemic to Australia and South America. Southern hemisphere taxa appear to have diverged more recently than the Northern hemisphere lineages. Our analysis of the *Tuberaceae* suggests that *Tuber* evolved from an epigeous ancestor. Molecular dating estimates *Tuberaceae* divergence in the late Jurassic (∼156 million years ago), with subsequent radiations in the Cretaceous and Paleogene. Intra-continental diversification, limited long-distance dispersal, and ecological adaptations help to explain patterns of truffle evolution and biodiversity.

## Introduction

Truffles are fungi that produce fruiting bodies with spores sequestered in a spherical mass, belowground or at the soil surface [1]. Many groups of truffles produce volatile aromatics. Although truffles have evolved in nearly every major group of fleshy fungi and over 100 times independently within the Ascomycota, Basidiomycota, and Mucoromycotina [2], the majority of transitions to a truffle form occur in ectomycorrhizal (EcM) fungal lineages [1]. This pattern suggests that the symbiotic association with plants may be an important driver in the evolution of truffle diversity. Truffles belonging to the *Tuberaceae*, such as the aromatic black truffle *Tuber melanosporum* Vittad. and white truffle *T. magnatum* Pico, have been collected and consumed by humans for centuries [3]. Despite this long history of human use, there are still many unanswered questions concerning the origin, historical biogeography, and ecology of these fungi.

**Figure 1:**
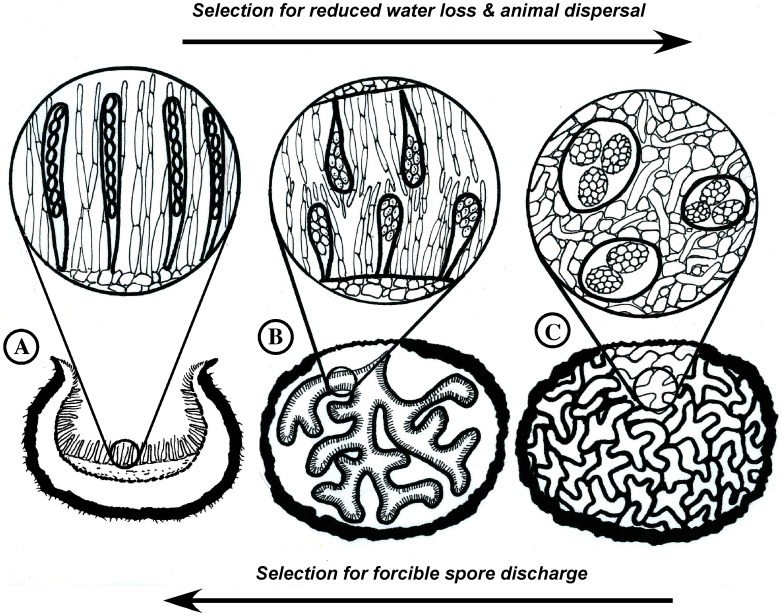
Hypothesized evolution of a truffle lineage. In this scenario the habitat of an epigeous species with 8-spored, uniseriate asci becomes more arid (A). Selection for reduced water loss results in an enclosed truffle form that has hymenium-lined chambers and asci that are shorter and more clavate in form (B). The ability to forcibly discharge spores is lost and selection for other means of spore dispersal intensifies, leading to spore dispersal through animal mycophagy. Continued selection results in a truffle species that fruits belowground and has a solid gleba stuffed with spherical asci packed with irregular numbers of spores (C).

The *Tuberaceae* are one of the most diverse lineages of exclusively truffle forming fungi [4] and are presumably one of the earlier diverging clades within the Pezizomycotina [5]. For instance, the genus *Tuber* (the most speciose of the five genera in the family) was estimated to comprise at least 180 species worldwide [6]. The loss of active spore discharge in fungi is correlated with the transition from an epigeous to hypogeous fruiting habit [4], [7]. Hypogeous fruitbodies of *Tuber* species are densely packed with spores produced in globose to subglobose asci (cells in which meiospores are formed) and veins of sterile tissue [8]. In contrast, epigeous relatives of truffles produce cup-shaped ascoma with elongated uniseriate asci capable of aerial spore discharge [9]. Given this drastic morphological differentiation, *Tuber* has been hypothesized to represent a “late-stage” in the evolutionary transition from an aboveground cup-fungus to a truffle (Fig. 1) [10]. The species *Tuber gennadii* (Chatin) Pat. is distinguished by the presence of locules lined by a palisade of asci and was hypothesized to be a transitional form between epigeous and hypogeous fruiting habits [11]. There are no true epigeous taxa known in the *Tuberaceae.*

The hypogeous fruiting habit of the truffle offers several selective advantages. Truffles are characterized by a low surface area-to-volume ratio, therefore a large number of spores are produced in a small packet of tissue. Furthermore, while epigeous fruiting bodies are directly exposed to weather, truffles are buffered against moisture and temperature fluctuations that might otherwise damage or inhibit development of spores. Truffle forming fungi have evolved novel mechanisms for spore dispersal via small animals that are correlated with the loss of active spore discharge. Many different animal species are attracted by the odors produced by truffle species [1] and truffle spores have been found in the fecal deposits of rodents, marsupials, reptiles and gastropods, suggesting that these animals are important dispersal agents of truffle spores [12], [13]. Indeed, truffle fruitbodies usually have durable, thick-walled spores that can withstand and possibly benefit from the passage through the digestive tract of animals [14]. The convergent evolution of these traits across a diversity of truffles lineages suggests that the transition from epigeous to hypogeous fruiting is driven by strong selection for traits that promote animal dispersal. Spore deposition via animal mycophagy may be a more targeted dispersal mechanism than wind or water dispersal [15], because animals that consume truffles are also likely to deposit their nutrient-rich and spore-laden fecal pellets near the roots of suitable host trees. Similarly, truffle consumption by highly dispersive animals may promote fungal colonization of new or distant habitats [16].

Loss of forcible spore discharge and adaptation to the hypogeous habit is often followed by extreme morphological changes, as seen in many different truffle lineages [1]. These morphological enigmas obscure taxonomic relationships between truffles and their epigeous relatives [1], [4]. Because morphological changes found in truffles appear to evolve rapidly, this form is likely due to the loss of function of a single or small set of genes that program the epigeous life history [17], [18]. Species in the *Tuberaceae* have undergone extensive morphological modifications compared to epigeous relatives within the Pezizales. For instance, species in the *Tuberaceae* typically have spherical or irregularly shaped asci and eight or often fewer ascospores per ascus. In contrast, epigeous species of *Pezizales* routinely have cylindrical asci with 8 spores per ascus (Fig. 1).

**Figure 2:**
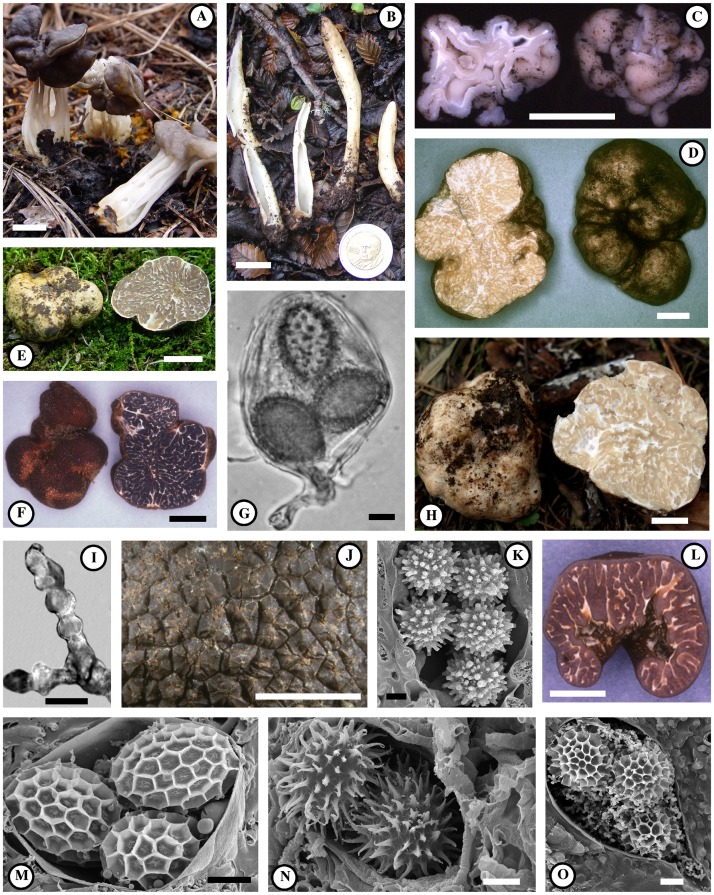
Morphological diversity and characters of truffles and their relatives. A. An “Elfin-saddle” cup-fungus *Helvella lacunose* Fr. Asci line the outside of the fertile cap, which is borne upon a stipe composed of vegetative tissue; B. the “earth-tooth” fungus *Underwoodia singer* Gamundí & E. Horak. A layer of fertile tissue lines the outside of the tooth-shaped cap. C. *Gymnohydnotrya* sp. collected under *Nothofagus pumilio* (Poepp & Endl.) Krasser in Argentina and similar to sequences from *Nothofagus* mycorrhizas. Fertile asci line both the inside and the outside the fruiting body. D. *Choiromyces alveolatus* (Harkn.) Trappe, a *Pinaceae* associate from western North America. E. A knobby-shaped representative of the/puberulum lineage, a clade of small, whitish truffles. F. *Tuber canaliculatum* Gilkey has a peridium covered in minute warts and its asci contain one or two reticulate spores. G. Flask-shaped ascus of the spiny-spored *Tuber lyonii* Butters with a stem at the point of attachment. H. Representative of the/japonicum lineage. I. Swollen beaded hyphae from the outer peridium of species belonging to the/gibbosum lineage. J. Large pyramidal warts cover the outer surface of *Tuber aestivum.* K. The spores of *Choiromyces meandriformis* Vittad. are ornamented with unusual pitted tubes. L. Species in the/excavatum lineage have a thick outer peridium and a partially enclosed internal cavity. M. Species in the/maculatum lineage have ellipsoid, alveolate-reticulate ascospores. N. The spores of *Tuber* sp.13 of the/melanosporum clade are particularly spiny. O. The spores of *Tuber spinoreticulatum* Uecker & Burds have spines that are irregularly connected by ridges that form a partial reticulation. Scale bars: A, B, C, D, E, F, H, J, L = 1 cm; G, I, K, M, N, O = 10 µm.

Previous phylogenetic studies of *Tuberaceae* have resolved two monophyletic Northern hemisphere clades, *Tuber* and *Choiromyces*, and a Southern hemisphere clade that includes *Dingleya, Reddellomyces,* and *Labyrinthomyces* [4], [7]. The sister group of the *Tuberaceae* remains unresolved [4]. The related *Helvellaceae*, previously regarded as the sister clade of the *Tuberaceae* (albeit without statistical support), are comprised of species producing either aboveground “elfin saddle” or sessile cup-shaped fruitbodies (e.g. *Helvella* – see Fig. 2a), or those with a truffle form (e.g. *Balsamia*) [4].

Dating the origin and diversification of fungi can be a contentious science, but methods for molecular dating are improving [19]. Because nucleotide substitution rates often differ between fungal lineages, a penalized likelihood method and fossil calibrations were used by Padovan *et al.* to estimate divergence dates within the Ascomycota based on a Bayesian phylogeny of 18S SSU rDNA [5]. They estimated the split of *Tuber* from other *Pezizales* occurred around 529 million years ago (Mya). In a more recent study focusing on the historical biogeography of *Tuber*, a molecular clock approach (with secondary calibration) was used to estimate the divergence times of major *Tuber* clades based on phylogenies inferred from multiple loci (18S rRNA, 5.8S-ITS2 rRNA, and ß-tubulin) [20]. Their results indicate that *Tuber* began to diverge during the Triassic or Jurassic between 271-140 Mya. However, these studies were limited by regional sampling and phylogenetic uncertainty, which may confound divergence time estimations.

Here, we estimate the phylogeny of a global sample of *Tuberaceae* employing both Maximum Likelihood (ML) and Bayesian inference methods based on DNA sequences of four genetic loci: ITS rRNA (ITS), 28S large subunit rRNA (LSU), elongation factor 1-α (EF1α), and RNA polymerase subunit II (RPB2). The main aims of this study were to: 1) estimate the phylogeny and divergence times for major clades of the *Tuberaceae*; 2) examine their major biogeographic patterns; 3) map characters to the phylogeny and reconstruct important ancestral morphological and ecological character states; and 4) determine their relationships to Southern hemisphere taxa of hitherto unknown phylogenetic affinities. We also used the expanded data set to test monophyly of the genus *Tuber*.

Because long-distance dispersal is often limited in fungi with hypogeous fruitbodies [21] we predicted that the biogeographic patterns of the *Tuberaceae* would fit a vicariance mode of distribution. Specifically, we hypothesized that 1) *Tuber* and *Tuberaceae* are monophyletic lineages composed strictly of truffle taxa; 2) most species and some lineages have restricted distributions at the continental scale, with major disjuncts between Northern and Southern hemisphere *Tuberaceae*; 3) spore ornamentation is a variable/plastic character that may vary between and (to a lesser extent) within *Tuber* clades; 4) divergence times of major clades within the *Tuberaceae* would track angiosperm radiations; and 5) inclusion of Southern hemisphere taxa would improve understanding of biogeographic patterns in the *Tuberaceae.*

## Materials and Methods

### Taxon Sampling

This global sampling of *Tuberaceae* integrated data from research programs in Europe [20], Asia [22], North America [6], [23] Central America, and South America, as well as extensive sampling of both public and private herbaria (Table S1). As outgroups, we used taxa belonging to the hypogeous genus *Balsamia* and epigeous genus *Helvella* [4] belonging to the *Helvellaceae* because these have been presumed to be the closest living relatives of the *Tuberaceae*. Our sampling includes representatives of *Tuber* previously analyzed in single locus (ITS) analyses [6] that comprised of 123 ITS phylotypes and represented approximately 70% of the accepted species, as well as 37 undescribed *Tuberaceae* species. We also included representatives from all the major *Tuberaceae* clades known from Japan and presented by Kinoshita et al. (2011). *Paradoxa* is a rare genus comprised of two species and is known only from the Northern Hemisphere (China and Italy). Although specimens of *Paradoxa* could not be obtained during the course of this study, specimens morphologically resembling *Paradoxa* were included [22]. We exclude taxa represented by only a single locus, with the exceptions of *Underwoodia columnaris* (which we included only to test the monophyly with Southern hemisphere taxa originally described as *Underwoodia*) and *Tuber sinoaestivum* and *Tuber* cf *excavatum* (which are the only known representatives of the/aestivum and/excavatum clades in Asia). Throughout this paper we adopt the use of rankless clade names following that of Moncalvo et al. [24], where the clade name is written in lowercase non-italicized letters and preceded with the symbol “/”.

### Molecular Data

Standard and touchdown polymerase chain reaction (PCR) protocols and fungal-specific primer sets (Table S2) were used to amplify and sequence four gene regions: the internal transcribed spacer ribosomal RNA gene (ITS), the 28S large subunit ribosomal RNA gene (LSU), elongation factor (EF1α), and the second largest subunit of RNA polymerase II (RPB2). EF1α and RPB2 could not be amplified for many *Tuber* species. To address this problem we designed a new set primers with enhanced specificity for the *Tuberaceae*: EF1α Tuber_f (5′ AGC GTG AGC GTG GTA TCA C 3′ – forward), EF1α Tuber_r (5′ GAG ACG TTC TTG ACG TTG AAG 3′ – reverse), and RPB2 Tuber_f (5′ Y AAY CTG ACY TTR GCY GTY AA 3′) paired with the reverse primer RPB2_Tuber_r (5′ CR GTT TCC TGY TCA ATC TCA-3′). Sequences produced for this study have been deposited in GenBank under the accession numbers JQ925626-JQ925656 (ITS), JQ925657-JQ925718 (LSU), JX022550-JX022615 (EF1α), and JQ954467-JQ954529 (RPB2) and alignments are available through TreeBASE (accession S13537). A complete list of specimens and sequences used in this study is provided in Table S1.

### Phylogenetic Reconstruction

Sequence alignments were initially performed in MUSCLE [25] individually for each locus. Alignments were visually inspected and ambiguous regions were excluded in Mesquite 2.5 [26]. Best-fit nucleotide substitution models were chosen through the Akaike information criterion, penalizing more complex models by one likelihood unit per additional free parameter, and ML phylogenetic trees for individual loci were estimated under these models in PAUP* [27]. Conflict among the four loci was assessed through strong incongruence of nodes based on 1000 ML bootstrap replicates (>70%) and posterior probabilities (>99%) of credible Bayesian trees. Because no strongly supported nodes were in conflict, the data sets were combined into a single matrix with four partitions. We conducted maximum likelihood (ML) and Bayesian inference (BI) analyses on individual and combined data sets. The ITS, LSU, EF1α, and RPB2 partitions included 274, 746, 813, and 735 characters, respectively, for a combined data matrix of 2568 characters. The number of included taxa were 99 (ITS), 96 (LSU), 80 (EF1α), and 67 (RPB2). Maximum likelihood analyses on the concatenated data were conducted with RAxML applying a GTRGAMMAI substitution model with parameters unlinked. ML bootstrap replicates (1000) were computed in RAxML under a GTRMIXI model, which infers an initial tree using the GTRCAT model, and then optimizes the tree topology using a GTRGAMMAI model. For Bayesian phylogenetic estimations, independent analyses were conducted with MrBayes [28]. Partitions were unlinked under either HKY+G+I (ITS and RPB2) or GTR+G+I (EF1α and LSU) nucleotide substitution models. Parallel runs with four chains were allowed to run 50,000,000 generations, sampling every 500 generations. Trees were sampled after the same likelihood plateau was reached between runs. MrBayes and RAxML analyses were computed through the CIPRES web portal (www.phylo.org).

In an attempt to better resolve the *Tuberaceae* a second round of analysis was performed with a more conservative alignment having fewer taxa and with/gymnohydnotrya as an outgroup. In these analyses introns within EF1α were excluded and amino acid positions were coded to compare alternative partition assignments. This more conserved alignment was also used in divergence time estimation analyses (below). Best-fit nucleotide substitution models were determined with PartitionFinder [29] under the Bayesian information criterion, which favors simpler models compared to the Akaike information criterion. The partitions included 40, 154, 77, 728, 635, and 735 characters for ITS1, 5.8 S, ITS2, LSU, EF1α and RPB2, respectively, for a combined data matrix of 2374 characters. Phylogenetic inferences were also conducted on a matrix consisting of 8 unlinked partitions: 1) SYM+I+G for 5.8S and RPB2 position 1; 2) JC for RPB position 2; 3) K80+I+G for RPB position 3; 4) F81+I+G for EF1α position 1; 5) JC+I+G for EF1α position 2; 6) GTR+G for EF1α position 3; 7) SYM+G for ITS1 and ITS2; 8) K80+I+G for LSU. Parallel runs with four chains were allowed to run 20,000,000 generations, sampling every 1000 generations.

### Divergence Time Estimation

**Figure 5:**
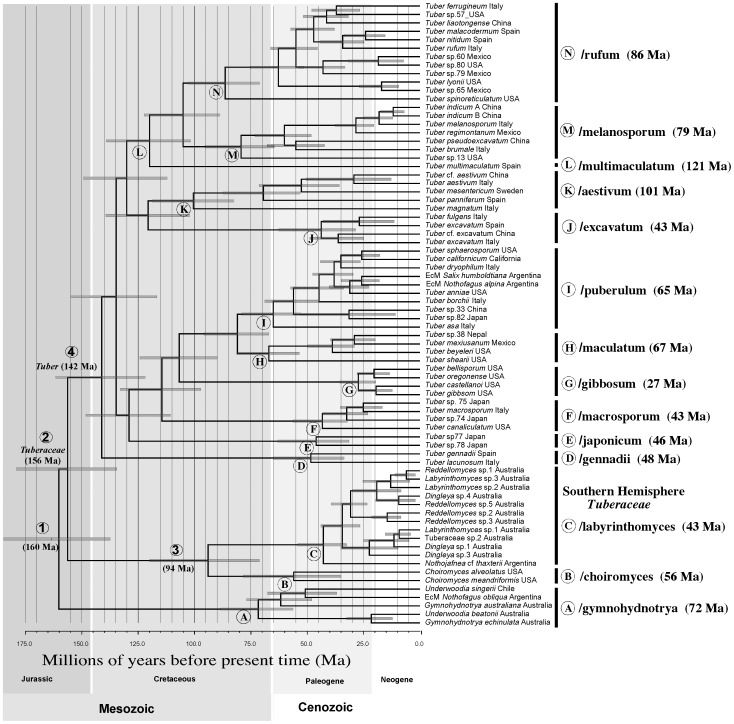
Bayesian Divergence Time Estimates for Truffles. The maximum clade credibility chronogram estimated in BEAST is shown with nodes placed at the median age. Node bars (grey) represent the node age 95% highest posterior density (HPD) for nodes receiving at least 50% Bayesian posterior probability. The median age is provided for labeled nodes (A–P) that are discussed in the text and node age parameters are presented in Table 1.

Molecular divergence time analyses were performed with the BEAST v1.7.2 software package [30] based on an alignment containing the four gene regions (ITS, LSU, EF1α, and RPB2) for a subset of the samples (one unique specimen per species – *see above*). Temporal calibration of divergence time analyses was achieved by fixing the absolute rate of molecular evolution for LSU locus (6.5×10^−4^ substitutions per site per million years) [31]. The evolutionary rates of the ITS, EF1α and RPB2 regions were estimated relative to the fixed LSU rate using a relaxed clock model with an uncorrelated exponential prior distribution with a mean of 1.0×10^−3^ substitutions per site per million years assigned to the mean rate of each region. It is important to emphasize that this prior is on the mean of the rate of each locus, and rate heterogeneity is modeled at each of these loci by an exponential distribution to avoid over constraining the rate and rate variation at these loci. Because all known *Tuberaceae* species are presumed to be ectomycorrhizal, we assume that the most recent common ancestor (MRCA) of these species was also ectomycorrhizal. Conservatively, we applied a maximum age constraint to the age to the MRCA of the *Tuberaceae* based on recent age estimates of the *Pinaceae* [32] (i.e. <250 million years ago), the oldest known lineage of obligate EcM hosts. A standard uniform prior, meaning an equal probability (i.e. flat distribution) between 0 and 250 Ma was applied to this node (node 2 in Fig. 5).

The sequence data were partitioned by gene region, with the exception of the ITS region, which was divided into three unique partitions: ITS1, 5.8S, and ITS2. The clock models and substitution models of the resulting six partitions were unlinked in BEAST analyses. The substitution models for the partitions were either HKY (RPB2) or GTR+G+I (LSU, ITS1, 5.8S, ITS2, EF1α) substitution models. We used gene partitions rather than codon positions because when data partitions become small the ability to estimate parameters for the substitution model or clock models suffers. The birth/death speciation model was employed, and a fully resolved starting tree was provided for each analysis. Three independent and identical BEAST analyses were each run for 30 million generations, sampling parameters and trees every 1000 generations. Parameters from the resulting 30 thousand generations for each of the three runs were examined for convergence, stationarity, and suitable effective sample sizes in the program Tracer v1.5 [33]. Based on this, a burn-in of 3000 trees was removed from each run, leaving 27,000 trees from each run, which were combined (81,000 trees) and used to generate a maximum clade credibility tree annotated with various parameter summary statistics using the program TreeAnnotator v1.7.2.

### Ancestral Character State Reconstructions

We reconstructed ancestral character states for EcM host plants of *Tuber* by phylum using the maximum likelihood model Markov k-state 1 parameter model in Mesquite [26]. Hosts were coded either as gymnosperms, angiosperms, or both (Table S1), considering a global database of *Tuber* ITS sequences that included host information were considered [6]. Ancestral state reconstructions were also carried out on fruitbody type (epigeous vs. hypogeous) in the *Tuberaceae* and/gymnohydnotrya using an asymmetrical 2-parameter Markov-K model. In this model, parameter values for the transition from an epigeous to hypogeous fruiting body were relatively high (≥10×) compared to the transition from hypogeous to epigeous fruiting habit, reflecting the reality that in nature forcible spore discharge is more easily lost than reacquired [21]. The program RASP [34] was used to statistically assess patterns of vicariance and dispersal across the genus using a distribution of equally probable Bayes trees and coding species by their geographical origins. Although *Tuber aestivum, T. excavatum, T. puberulum, T. oligospermum* and *T. rufum* have been reported from Northern Africa [20], collections from Africa were not available for study, and consequently, we did not include this biogeographic region in our analyses.

## Results

### Phylogenetic Analyses

**Figure 3:**
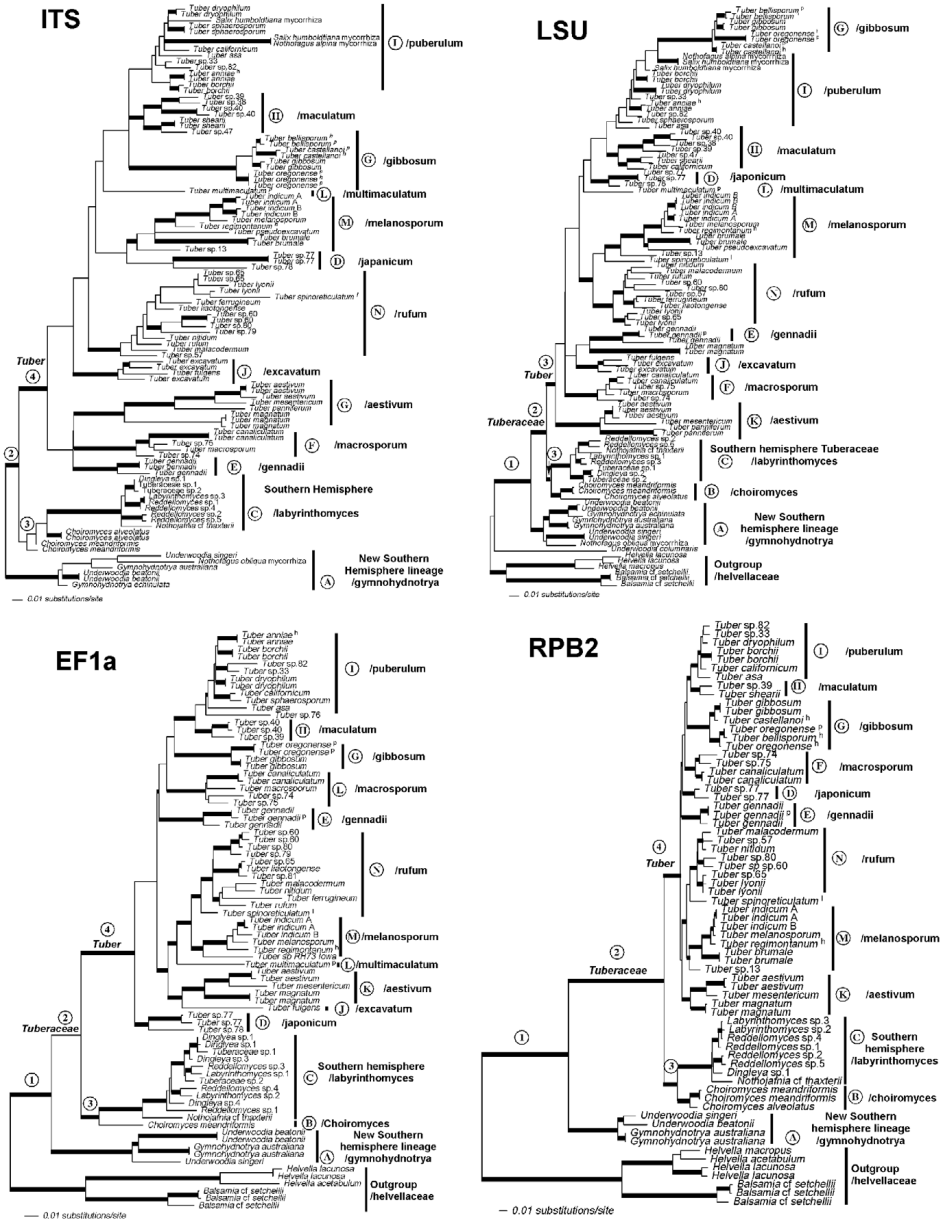
Phylogenetic reconstructions of *Tuber* based on maximum likelihood analysis of four individual loci: internal transcribed spacer region (ITS), 28 **s large subunit rDNA (LSU), elongation factor 1-alpha (EF1a), and RNA polymerase II (RPB2).** Models and likelihood scores for each locus are: ITS = Sym +G+I (–3960.627); LSU = GTR +G+I (−8732.114); EF1a = GTR +G+I (7374.012); RPB2 = K80 (5880.021). Clade names and node labels are consistent with each other and with figures 4 and 5. Taxa in the *Helvellaceae* were excluded from the ITS analysis because of the alignment challenges imposed by sequence divergence.

**Figure 4:**
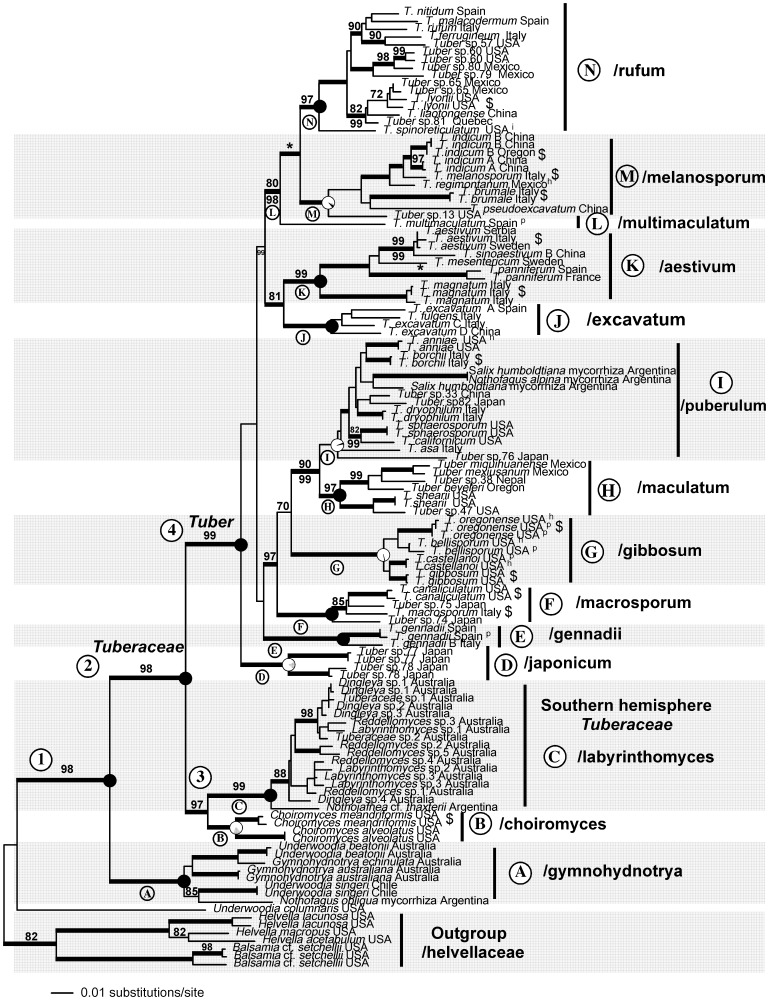
Maximum likelihood (ML) phylogenetic reconstruction of the *Tuberaceae* phylogeny based on ITS, 28S rDNA, EF1α, and RPB2 gene regions. Thickened branches represent ML bootstrap support >70 and posterior probabilities of 100. ML bootstrap values above nodes are based on 1000 replicates. Posterior probabilities are presented below nodes. Thickened branches without numbers received maximum ML and Bayesian support values. Reconstructed ancestral host plant associations (based on maximum likelihood) are represented at internal nodes by circles; black for ancestors in symbiotic association with angiosperms, white for ancestors in symbiotic association with *Pinaceae*, and gray for ancestors in symbiotic association with angiosperms and *Pinaceae*. Nodes supported by transitions in spore ornamentation from alveolate-reticulate to spiny are shown with an asterisk *. Economically important species are denoted by the symbol $ after their name and geographic origin. The phylogeny is rooted with taxa from the *Helvellaceae* including species of epigeous *Helvella* and hypogeous *Balsamia*. Major lineages of *Tuber* and *Tuberaceae* are indicated to the right of the tree. The *Tuberaceae* form a monophyletic group, which is resolved as a sister group to a previously unrecognized Southern hemisphere lineage (/gymnohydnotrya). Type specimens are denoted by the superscripts: ^h -^ holotype, ^i -^isotype, ^p -^paratype.

Individual loci (Fig. 3) and combined molecular data (Fig. 4) confirm that the *Tuberaceae* is monophyletic as are both of the Northern hemisphere genera *Tuber* and *Choiromyces*. In contrast, genera of Southern hemisphere *Tuberaceae* were not resolved as monophyletic and are in need of taxonomic revision. Phylogenies of *Tuber* based on individual loci reconstructed the same major clades (Fig. 3). However, the LSU phylogeny does not resolve the/puberulum lineage or place *T. magnatum* within the/aestivum lineage. Because there was no strongly supported conflict between single gene phylogenies, we combined the data sets to improve phylogenetic resolution. Eleven major clades can be recognized within *Tuber* based on the concatenated dataset (Figs. 4 & 5). The/rufum,/melanosporum,/puberulum,/maculatum, and/macrosporum clades are distributed across the entire Northern hemisphere (Europe, Asia, North America, Central America and Northern Africa), yet are characterized by a high degree of species-level endemism. On the other hand, several *Tuber* clades are endemic to particular continents:/gennadii and/multimaculatum to Europe,/japonicum to Asia, and/gibbosum to North America. The/aestivum and/excavatum groups are distributed across Europe and Asia. Economically important *Tuber* species are interspersed within six of the eleven major clades in Europe, Asia, and North America (Fig. 4, Table S3).

A number of noteworthy discoveries came from our inclusion of Southern hemisphere taxa from South America (7) and Australia (21). First, we sequenced two novel *Tuber* species from multiple root samples collected in Argentina, indicating that *Tuber* is not strictly a Northern hemisphere genus. These sequences were placed in the/puberulum lineage and were derived from ectomycorrhizas sampled in natural stands of *Nothofagus* spp. and *Salix humboltiana* Willd., both native to South America [35]. These findings support the anomalous report of a native Argentinean *Tuber* species, *T. australe* Speg. [36]. Second, we demonstrate that the epigeous South American cup-fungus, *Nothojafnea thaxteri* (Cash) Gamundí, represents an early diverging lineage within the *Tuberaceae* and is closely related to the Australian truffle genera *Reddellomyces*, *Labyrinthomyces*, and *Dingleya* (Fig. 4). Our findings show that *N. thaxteri* is the closest known extant epigeous relative of the genus *Tuber*. This is the first report of an epigeous (non-truffle) species in the *Tuberaceae* sensu stricto. Third, we identified a previously unrecognized Southern hemisphere clade, which is supported as the sister group to the *Tuberaceae*. This clade (/gymnohydnotrya) is known from South America and Australia and contains taxa that form either epigeous (*Underwoodia pro parte)* or hypogeous (*Gymnohydnotrya*) fruitbodies. Our phylogenetic treatment of *Nothojafnea* and/gymnohydnotrya constitutes the first evidence that these taxa are related to *Tuberaceae*.

### Estimated Divergence Times

Median date estimates for the origin of the *Tuberaceae,* based on a maximum age constraint of <250 Mya, are in the late Jurassic (Fig. 5 - node 2) at 156 million years ago (Mya). We estimated that *Tuber* diverged from other genera in the early Cretaceous 156 Mya (Fig. 5 - node 4), and by end of the Cretaceous (65 Mya) most of its extant subgeneric lineages were present. However, major radiations within these lineages occurred during the Paleogene (Fig. 5). The divergence time estimates and confidence intervals are summarized for *Tuber* clades in Table 1. Estimates for the mean ages of the MRCA of *Tuber* clades (Fig. 5) are:/multimaculatum (121 Mya),/aestivum (101 Mya),/rufum (86 Mya),/melanosporum (79 Mya),/puberulum (65 Mya),/japonicum (46 Mya),/excavatum (43 Mya),/maculatum (67 Mya),/macrosporum (43 Mya),/gennadii (48 Mya), and/gibbosum (27 Mya). Our age estimates for MRCA of *Tuberaceae* and its newly recognized sister lineage (/gymnohydnotrya – Fig. 5 - node 1) was 160 Mya, which corresponds to the late Jurassic. The estimated divergence of the/labyrinthomyces lineage (Fig. 5 - node C) at 43 Mya is relatively recent compared to *Tuber*.

**Table 1:** Divergence time estimates (in millions of years) from shared common ancestors for major clades within *Tuberaceae* as referred to in Figures 3–5.

Clade (Ma)	Node	Median Age (Ma)	Age 95% HPD
/gymnohydnotrya	A	72.0	56.3–88.4
/choiromyces	B	56.8	35.3–78.3
/labyrinthomyces	C	43.2	32.6–54.2
/japonica	D	46.6	31.6–63.1
/gennadii	E	49.0	33.6–65.2
/macrosporum	F	43.7	32.2–56.3
/gibbosum	G	27.6	19.7–35.7
/maculatum	H	67.4	53.5–81.3
/puberulum	I	65.4	52.9–79.0
/excavatum	J	45.2	28.5–62.7
/aestivum	K	69.8	53.1–87.3
/melanosporum	L	79.7	64.6–95.4
/rufum	M	86.8	71.2–103.0
/tuberaceae −/gymnohydnotrya	1	160.8	137.4–184.7
/tuberaceae	2	156.9	134.5–179.1
/choiromyces −/labyrinthomyces	3	94.9	71.2–120.0
/tuber	4	141.6	121.8–161.6

Nodes D-M represent major genetic groups within *Tuber*.

Evolutionary rates of the ITS1, 5.8S, ITS2, EF1a and RPB2 regions were calculated relative to fixed LSU rates using a relaxed clock model with uncorrelated exponential prior distributions (see methods). Our mean posterior rate estimates in substitutions per site per million years for the specific partitions are as follows: ITS1 = 1.72E-3; 5.8S = 3.02E-4; ITS2 = 2.07E-3; EF1a = 4.01E-4; RPB2 = 3.78E-4.

### Ancestral Character State Reconstructions

Extant species in a number of *Tuber* clades can associate with angiosperms, *Pinaceae*, and parasitic orchid monocots (e.g. *T. aestivum* Vittad.) [6]. Character state reconstructions indicate the most recent common ancestor to the *Tuberaceae* was likely an ectomycorrhizal symbiont of angiosperms. There appear to be multiple independent shifts to *Pinaceae* hosts, particularly at the nodes of the/gibbosum,/melanosporum, and/puberulum clades. Divergence date estimations (reported above) place the MRCA of both the *Tuberaceae* and/gymnohydnotrya lineage in the early age of angiosperms, yet it is possible that these ancestral species were mycorrhizal with *Pinaceae*, or lived as endophytes, pathogens or saprotrophs.

Spore ornamentation is one of the most important characters for truffle taxonomy. Consequently, we were interested in reconstructing the evolution of spore characters in the genus *Tuber.* Spores of *Tuber* species are either ornamented with an alveolate-reticulate pattern (e.g. honeycomb design – Fig. 2 M), spines (Fig. 2 N), or spino-reticulation (e.g. spines connected by ridges Fig. 2 O). Based on our analyses, alveolate-reticulate ornamentation is the plesiomorphic (ancestral) condition for *Tuber*. At least two independent transitions from alveolate-reticulate to spiny ornamentation have occurred: one in the ancestor of the/melanosporum−/rufum lineage, and another in the ancestor of *T. panniferum* Tul. These are depicted by an asterisk (*) above the nodes of these clades in figure 4. Several apparent reversals from spiny to alveolate-reticulated spores have also occurred (e.g. *T. liaotongense, T. pseudoexcavatum*).

Fungal fruiting bodies can have diverse forms. The epigeous *Nothojafnea* cf. *thaxterii* specimen occurs within the *Tuberaceae*, a family historically considered to contain strictly hypogeous taxa. Moreover, the/gymnohydnotrya and/labyrinthomyces lineages include both epigeous and hypogeous species. Accordingly, ancestral state reconstructions of fruitbody habit in the *Tuberaceae* were conducted to determine how many transitions to a belowground fruiting habit occurred in this lineage. Unweighted parsimony and single parameter likelihood models indicated that the ancestor to *Tuberaceae* was hypogeous and that a single transition to an epigeous fruiting form occurred in the ancestor of *Nothojafnea.* However, as mentioned previously, the ability to regain forcible spore discharge is considered highly unlikely in fungi and we know of no unequivocal cases where this has been previously shown. Using a 2-parameter model we found that models with forward to reverse (epigeous → hypogeous) transition ratios of 10∶1 or greater reconstruct the ancestor of *Tuber* as most likely epigeous. Similarly, the ancestor of the/labyrinthomyces lineage and/labyrinthomyces-choiromyces lineages are reconstructed as epigeous.

**Figure 6:**
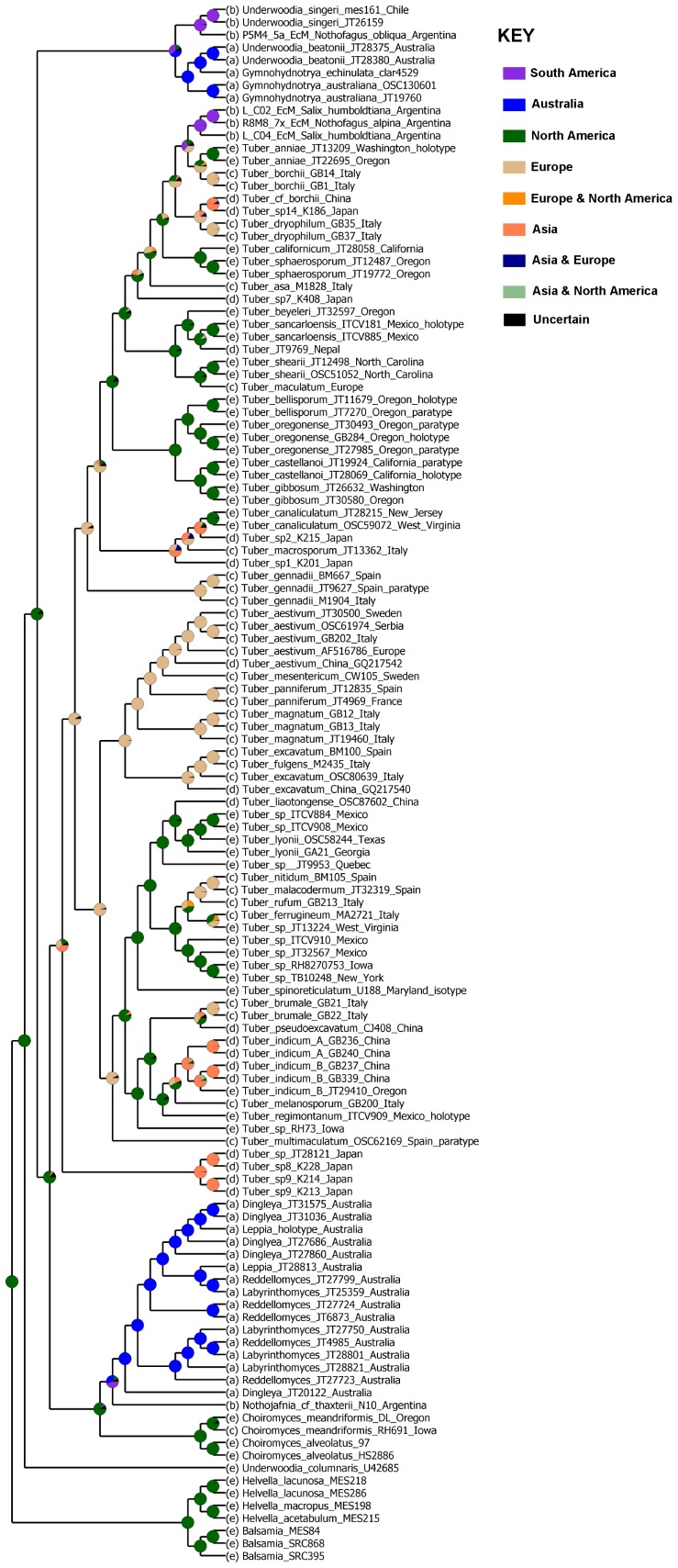
Results of statistical dispersal-vicariance (S-DIVA) analyses of the *Tuberaceae.*

Inter-continental dispersal is evident in most major *Tuber* clades. Our results indicate that vicariance alone cannot explain the modern distribution of extant *Tuberaceae* (Fig. 6). Although there is still uncertainty concerning the origin of *Tuber*, putative geographical origins of the most common ancestors for most clades can be resolved. North America appears to be the ancestral area of the/gibbosum,/maculatum,/rufum, and/melanosporum clades, whereas Europe is likely the ancestral area of the/aestivum,/excavatum, and/gennadii clades. Asia is ancestral area for the/japonicum lineage.

## Discussion

### Multiple Independent Evolutionary Transitions to the Truffle form in the *Tuberaceae*

Truffles are derived from aboveground fruiting ancestors, however, historically the family *Tuberaceae* has been regarded to be composed of strictly hypogeous species [4], [7]. We show here for the first time that the Argentinean cup-fungus *Nothojafnea thaxteri* (Cash) Gamundí is the earliest diverging member in the Southern hemisphere/labyrinthomyces lineage, and the only known epigeous species that can be placed within the *Tuberaceae*. We infer from asymmetric 2-parameter ancestral state reconstructions that at least three transitions to belowground fruiting have occurred within the *Tuberaceae*: one leading to the rest of the/labyrinthomyces lineage, a second transition leading to the genus *Choiromyces,* and a third transition leading to *Tuber*. Multiple independent transitions to a truffle fruiting habit are also evident in/gymnohydnotrya, the sister lineage of the *Tuberaceae*. These truffles appear to be derived from an epigeous “earth-tooth” fungus, with affinities to *Underwoodia singeri* Gamundí & E. Horak (Fig. 2B).

### Historical Biogeography of *Tuber*

Our data provide high statistical support for the monophyly of *Tuber* and *Tuberaceae,* in agreement with our initial hypothesis and previous studies [6], [20], [23]. We estimate that *Tuber* began diverging in the early Cretaceous, around 142 Mya (Fig. 5 - node 4), which would coincide with the emergence of Eudicots and near complete tectonic breakup of Pangea [37]. This date is also within the range estimates of 140–271 Mya calculated by Jeandroz et al. [20] using molecular clock approaches of ribosomal and beta-tubulin genes. Overall, the divergence date estimates for commonly recognized *Tuber* clades by Jeandroz et al. [20] were younger for the shallower nodes and older for deeper nodes compared with our divergence date estimates. These discrepancies are likely due to differences in taxon sampling, phylogenetic resolution, and methods for dating divergence times. In particular, Jeandroz et al. [20] assumed a linearized tree approach and a single fixed calibration point meaning that topological and branch length uncertainty are not accounted for in their divergence time estimates. In contrast, Bayesian methods developed and used here are better able to deal with this uncertainty [30]. Further, we have included a more thorough phylogenetic sampling within the family *Tuberaceae*, the genus *Tuber*, and within each of the major *Tuber* clades leading to a more complete and resolved model for the phylogenetic structure of this family.

Our synthesis confirmed relationships among major *Tuber* clades that were detected in previous studies [6], [20], [22], [23] but we also uncovered new biogeographical patterns such as the occurrence of *Tuber* in South America. Global diversity of *Tuber* species is high, and this may be partly due to a high level of regional endemism [6], [22], [38]. For instance, Kinoshita et al. [22] recently reported more than 20 undescribed *Tuber* species in Japan, including members of the/japonicum lineage. The addition of phylogenetically dispersed representatives of these species provided insights on the historical biogeography of *Tuber*, but the relationship between the/japonicum and/gennadii lineages to the rest of *Tuber* was still not fully resolved with this dataset.

While we must reject a strict vicariance model for explaining the biogeography of *Tuber*, particularly for the/puberulum group that is the most widely distributed clade, most species and many *Tuber* clades appear to have restricted distributions at the continental scale in accordance with our hypothesis. For instance, the/gibbosum,/japonicum,/gennadii, and/multimaculatum clades appear to be restricted to single continents. Consistent with a vicariance model of diversification, Europe and Asia (which have had greater geographic connectivity) share more *Tuber* lineages (but not species) with each other (e.g./excavatum and/aestivum) than they do with North America. However, some lineages (i.e./rufum,/melanosporum, and/macrosporum) are distributed across Europe, Asia, and North America, indicating past dispersal (or migration) between the continents, putatively via the Beringia Land Bridge (between North America and Asia) and the Thulean North Atlantic Land Bridge (between Europe and North America). Major disjuncts were observed between the (almost entirely) Northern hemisphere *Tuber* and Southern hemisphere/labyrinthomyces and/gymnohydnotyra lineages. Our date estimates of the divergence of these lineages (156–160 Mya) correspond well with the early splitting of Gondwana and Laurasia, except in the case of/choiromyces. Our data indicates that/choiromyces diverged from/labyrinthomyces 94 Mya, well after the split of Gondwana and Laurasia, thus dispersal must be invoked to explain this biogeographic distribution.

We estimate that the most recent common ancestor of the/japonicum clade radiated around 46 Mya (Fig. 5 - node D) in Asia. Species in the/japonicum group are light in color and have irregularly alveolate-reticulated spores that are pale yellow at maturity [22]. Also, species in this clade tend to have only one or two spores per ascus, fewer than most other *Tuber* species.

The/gennadii lineage is another early diverging clade within *Tuber.* This group appears restricted to Europe. There has been much confusion regarding the taxonomy of the species within this clade. Originally described as *Terfezia gennadii* by Chatin in 1896, Patouillard transferred this species to *Tuber* in 1903. More recently Alvarez et al. (1992) placed this species into the monotypic genus *Loculotuber* because of its distinct morphology of chambers (locules) lined with fertile asci. However, our data place it as a distinct clade within the genus *Tuber*. Alvarado et al. [39] have identified two species in this clade (*T. gennadii* and *T. lacunosum*) and we estimate that their most recent common ancestor radiated in Europe around 48 Mya (Fig. 5 - node E) in association with angiosperm hosts.

*Tuber multimaculatum* Parladé, Trappe & I.F. Alvarez is the sole representative in the/multimaculatum lineage (Fig. 5 - node L), and is only known from a few collections [11]. Possibly due to its long branch on the phylogeny, its exact placement within the genus *Tuber* differs depending on which gene is used to reconstruct the phylogeny. *Tuber multimaculatum* was estimated to have shared a common ancestor with other *Tuber* species 121 Mya (Fig. 5 - node L). Distinctive features of *T. multimaculatum* include 1-spored or 2-spored asci that have notable apical thickenings in the ascus walls, as well as large ellipsoid ascospores that have finely meshed alveolate reticulations.

The/macrosporum lineage is characterized by the presence of small warts on the outside surface of the peridium and typically 2- or 3-spored asci containing relatively large alveolate-reticulate spores. We show for the first time that this group occurs in Asia, Europe, and North America. Some species in this group are associated with angiosperm hosts, but others are associated with species of *Pinaceae*. We estimate that the most recent common ancestor of this clade radiated in Europe around 43 Mya (Fig. 5 - node F) but the geographical origin and ancestral host group were poorly resolved.

The/gibbosum lineage is composed of four species of light-colored truffles that are characterized by beaded hyphae (Fig. 2I) that emerge from their peridia [23]. The/gibbosum lineage is unique in that species in this clade appear to associate exclusively with *Pinaceae* hosts, particularly with *Pseudotsuga* but also with *Pinus* [23], [40]. This lineage is restricted to western North America and our molecular dating results indicate that the most recent common ancestor of this clade radiated in the Western North America around 27 Mya (Fig. 5 - node G) in association with *Pinaceae*. Estimated dates for the radiation of species with the/gibbosum lineage correspond closely with the estimated age of the *Pseudotsuga* radiation in western North America (∼22 Mya) [41]. We hypothesize that the transition to a conifer host may have facilitated species diversification within this *Tuber* lineage.

The/maculatum lineage is composed of light-colored truffle species that have a smooth to cracked outer peridium and elliptical alveolate-reticulate ascospores [42]. The majority of species in this lineage are undescribed, but they appear to be associated with angiosperm hosts and are mainly distributed in North America and Europe [6]. We estimate that the most recent common ancestor of this clade radiated in North America around 67 Mya (Fig. 5 - node H) in association with angiosperm hosts. Jeandroz et al. [20] calculated a similar divergence date (65 Mya) at this node based on molecular clock analysis of 5.8S and ITS2 rDNA.

The/puberulum lineage is one of the most diverse in *Tuber.* Species in this clade produce light-colored truffles that have a smooth to cracked peridium and globose to subglobose ascospores with alveolate-reticulation. The multigene phylogeny (Fig. 4), phylogenetic trees based on individual loci (Fig. 3), and previous published studies [6], [20] recover the/puberulum clade, but bootstrap support values are low. Species in this clade are distributed across Europe, Asia, North America, South America, and northern Africa and they are found in association with *Pinaceae*, angiosperms, or both. The two South American species included in this study were recovered from ectomycorrhizas (Nouhra et al., unpublished) and were placed on a long branch in the phylogram (we were only able to amplify ITS and LSU from these root tips, despite multiple attempts to amplify other loci). Although ectomycorrhizae of the European species *T. melanosporum* have been formed on *Nothofagus* in a greenhouse [43], this is the first evidence of a *Tuber* species from natural stands of *Nothofagus*. Many *Tuber* species in the/puberulum clade are known to associate with *Salix* spp. [6], but we are not able to determine at this time whether these *Tuber* species tracked the migration of *Salix* to South America, or whether these *Tuber* species were present in South America prior to the immigration of *Salix* (e.g. associated with *Nothofagus*). We estimate that the most recent common ancestor of the/puberulum clade diverged 65 Mya (Fig. 5 - node I) but their geographical origin and ancestral host group were poorly resolved. Our results indicate that species in the/puberulum lineage are well adapted for long-distance dispersal compared to other *Tuber* clades. For instance, they are the only group of *Tuber* naturally represented in the Southern hemisphere (e.g. Argentina), but dispersal by ship on roots of seedlings of European and North American mycorrhizal host trees is likely for the species reported for New Zealand [44]. Jeandroz et al. [20] calculated the divergence date of this node (Fig. 4 - node I) at 33 Mya based on molecular clock analysis of 5.8S and ITS2 rDNA.

Species in the/excavatum lineage are characterized by a basal cavity, a thick and hard peridium, and coarsely reticulated ascospores. They are symbionts of angiosperms and are distributed in both Europe and Asia. We estimate that the most recent common ancestor of this clade radiated in Europe or Asia around 43 Mya (Fig. 5 - node J) in association with angiosperms. This clade also appears to contain many cryptic species sharing similar morphology [6].

The/aestivum lineage is also distributed across Europe and Asia. Species in the group occur mostly in association with angiosperms, although *T. aestivum* may also associate with some *Pinaceae* hosts. This clade is characterized by the highest level of morphological diversity of the genus and appears to have been among the first *Tuber* clades to diversify. We estimate its most recent common ancestor radiated in Europe around 101 Mya (Fig. 5 - node K) in association with angiosperms. Jeandroz et al. [20] calculated the divergence date of this node similarly at 70 Mya based on molecular clock analysis of 5.8S and ITS2 rDNA.

The/melanosporum lineage is distributed across Europe, Asia and North America. Most species in this clade are characterized by large peridial warts and darkly pigmented ascospores ornamented with spines that sometimes connect to form a reticulum. We estimate that the most recent common ancestor of this clade radiated in association with *Pinaceae* in North America around 79 Mya (Fig. 5 - node M) followed by subsequent dispersal events to Asia and Europe. Jeandroz et al. [20] calculated the divergence date of this node at 76 Mya based on molecular clock analysis of 5.8S and ITS2 rDNA.

The/rufum lineage is well supported as the sister group to the/melanosporum lineage and is also distributed across Europe, Asia and North America. Species in the/rufum lineage are primarily found with angiosperm hosts [6] and are characterized by a smooth to minutely warted peridium with light-colored ascospores ornamented with spines. In a few species, such as in *T. spinoreticulatum*, the spines may connect to form a partial reticulum (Fig. 2O). The most recent common ancestor of this clade was estimated to have radiated in North America around 86 Mya (Fig. 5 - node N) in association with angiosperm hosts. This lineage later dispersed to Asia and Europe. Jeandroz et al. [20] calculated the divergence date of this node at 70 Mya based on molecular clock analysis of 5.8S and ITS2 rDNA.

### *Tuberaceae* Radiated with Angiosperm Host Plants during the Cretaceous and Paleogene

The *Tuberaceae* are presumed to share an ancient ectomycorrhizal ancestor because this is the nutritional mode for all of the extant species [4]. Alternatively, the ectomycorrhizal habit may have been acquired independently in the *Tuberaceae*,/gymnohydnotrya clade, and *Helvellaceae*, but this seems unlikely given that no saprotrophic fungi are documented for any of these three lineages. Northern hemisphere *Tuberaceae* species are associated with a wide diversity of host plants including both monocot and dicot angiosperms and *Pinaceae*, but their ancestral ectomycorrhizal hosts are unknown. In accordance with our hypothesis, ancestral state reconstructions recover the ancestor of *Tuber* (and *Tuberaceae*) as most likely ectomycorrhizal with angiosperm hosts (Fig. 4 - nodes 4, 2). The hypothesis of an ancient symbiotic association between *Tuberaceae* and angiosperm host plants is also supported by the fact that extant members of the/labyrinthomyces and the/gymnohydnotrya lineages are exclusively associated with angiosperms (they occur in the Southern hemisphere where *Pinaceae* do not naturally occur). Species of the Northern hemisphere genus *Choiromyces* may associate with either angiosperms or *Pinaceae* [45], [46], [47]. It appears that multiple independent ecological transitions from angiosperm to *Pinaceae* hosts have also occurred in individual *Tuber* species (e.g. *T. canaliculatum* Gilkey, *T. indicum* Cooke & Massee, *T. borchii* Vittad.) and for entire clades (e.g./gibbosum). We find it interesting that many clades of *Tuber* have species susceptible to orchid parasitism [6], [48], which raises many questions pertaining to plant-fungus interactions.

Our molecular dating results place the *Tuberaceae* origin at the end of the Jurassic period (156 Mya), during the early radiation of angiosperm Eudicots [49], and are thus congruent with our hypothesis that the *Tuberaceae* initially co-radiated with angiosperm hosts. Although most of the major *Tuber* clades had evolved by the end of the Cretaceous, radiations within most species-rich lineages occurred during the mid-Paleogene (30–54 Mya), a time when angiosperms, *Pinaceae*, and other plant-associated fungi were all experiencing major evolutionary radiations [50]. These dates generally correspond to estimated radiations in major ectomycorrhizal host plant lineages including the *Fagaceae*, *Betulaceae*, *Salicaceae* and *Juglandaceae* [51], [52], [53], [54]. We posit that the diversification of ectomycorrhizal angiosperm hosts during this period may have driven the diversification within *Tuber* and possibly other ectomycorrhizal lineages [55].

Other studies have shown that many other fungal groups were undergoing radiations during the Cretaceous period. For instance, Matheny et al. [56] used a relaxed molecular clock multi-locus approach to study the historical biogeography and diversification of a family of ectomycorrhizal basidiomycetes, the *Inocybaceae*. Their analyses indicate that the major clades within this family diverged during the Cretaceous (143 Mya) in association with angiosperms. The genera *Amanita* and *Hygrophorus* also likely have Cretaceous crown group origins [55]. O’Donnell et al. [57] studied the historical biogeography of the true morels (*Morchella*), a saprotrophic and biotrophic ascomycete lineage that is related to the *Tuberaceae*, and they used a multi-locus strict molecular clock approach. They found that *Morchella* diverged from its closest relatives in the early Cretaceous (126.6 Mya) and exhibited high continental endemism and provincialism. In another study, Sung et al. [58] examined fungal-animal symbionts in the Hypocreales using a Bayesian relaxed molecular clock approach and a fossil from the lineage as a calibration point. Their results indicated that the major families within the Hypocreales all diverged during the Cretaceous. Thus, many fungal groups appear to have undergone radiations during this geological period.

### Other Lineages in the Tuberaceae

*Choiromyces* is a monophyletic genus broadly distributed in the Northern hemisphere. However, it appears more closely related to the Southern/labyrinthomyces lineage than to *Tuber* (Fig. 4 - node 3). The hypogeous fruitbodies of *Choiromyces* are subglobose or irregular in form (Fig. 2D) and are characterized by a solid gleba having a hymenium with paraphyses and clavate asci usually bearing eight-spores [59]. These ascospores have distinct ornamentation of either pits or pitted tubes (Fig. 2K).

The Southern hemisphere *Tuberaceae* also form a monophyletic group, the/labyrinthomyces lineage. Taxa include both truffle and the cup-shaped forms. The cup fungus *Nothojafnea thaxteri* has 8-spored, cylindrical asci with uniserate spores whereas the truffle genera *Dingleya*, *Labyrinthomyces*, and *Reddellomyces* are morphologically diverse. They can have between one to eight ascospores, their asci can be uniseriate, cylindrical, or saccate, and they have widely diverging peridial morphologies [60], [61], [62]. However, the generic boundaries between these truffle genera are not particularly clear since morphological characters are not consistent with the phylogeny (Fig. 4). We estimated relatively short divergence times between taxa in the/labyrinthomyces lineage, which may explain some of the taxonomic problems with this group.

### Enigmatic Taxa – Nothojafnea, Gymnohydnotrya, and Underwoodia

As we initially hypothesized, the inclusion of Southern hemisphere taxa contributed greatly to a better understanding of the *Tuberaceae*, to the identification of its sister lineage, and provided novel data concerning their evolution and biogeography. *Nothojafnea*, *Gymnohydnotrya*, and *Underwoodia* are three genera of enigmatic *Pezizales* whose phylogenetic positions are poorly known. Our phylogeny shows for the first time that *Nothojafnea* and *Gymnohydnotrya* are affiliated with the *Tuberaceae*. We also confirmed that the genus *Underwoodia* is polyphyletic. The North American type species, *U. columnaris,* is allied with *Helvella* and *Balsamia* in the *Helvellaceae,* whereas the two Southern hemisphere species, *U. singeri* (South America) and *U. beatonii* Rifai (Australia) are allied with *Gymnohydnotrya.* Together these Southern hemisphere species form a previously unrecognized sister group to the *Tuberaceae*,/gymnohydnotrya, which will be formally described and named in a separate paper. Moreover, *U. singeri* and *U. beatonii* are not sister species and it is likely that *Gymnohydnotrya* truffles have evolved multiple times within the Southern hemisphere “*Underwoodia”*.

*Nothojafnea* is one of the enigmatic ectomycorrhizal genera whose taxonomic placement has long remained a mystery [2]. Only two species are described in the genus, *Nothojafnea cyptotricha* Rifai from Australia and *Nothojafnea thaxteri* (E.K. Cash) Gamundi from Argentina and Chile [63], [64]. Based on the ornamented spores and prominent apothecial hairs, the genus was described in the family *Pyronemataceae* [64]. There is strong support for the placement of *N. thaxteri* in the/labyrinthomyces lineage and sister to the Australian truffle genera *Dingleya*, *Labyrinthomyces*, and *Reddellomyces* (Fig. 5 - node C). Both species of *Nothojafnea* are considered ectomycorrhizal symbionts since they fruit directly on soil beneath ectomycorrhizal plants. The holotype species, *N. cryptotricha,* is found with *Myrtaceae* genera including *Eucalyptus* and *Melaleuca* [64], [65] whereas *N. thaxteri* has only been found with *Nothofagus* [66]. Warcup [65] provided further verification of the symbiotic ecology of *Nothojafnea* when he synthesized ectomycorrhizas of *N. cryptotricha* in pot cultures with *Melaleuca uncinata* R. Br.

As with *Nothojafnea*, the taxonomy and ecology of the genus *Gymnohydnotrya* is poorly known [2]. *Gymnohydnotrya* originally accommodated three Australian truffle species, *G. australiana* B.C. Zhang & Minter, *G. echinulata* (G.W.Beaton) B.C. Zhang & Minter, and *G. ellipsospora* (J.W. Cribb) B.C. Zhang & Minter [67]. The genus is characterized by light colored ascomata with an externally facing hymenium (exothecium), a basal hyphal tuft and no peridium. Microscopically, *Gymnohydnotrya* species tend to have 8-spored asci and hyaline, ornamented ascospores [67]. *Gymnohydnotrya* species are considered ectomycorrhizal because they fruit in soil and leaf litter beneath *Eucalyptus* and other Australian ectomycorrhizal plants [2], [67], [68]. Here an isolate from healthy root tips of *Nothofagus obliqua* was strongly supported as a member of the/gymnohydnotrya lineage, providing the first direct evidence for its ectomycorrhizal lifestyle. Although there are currently no species of *Gymnohydnotrya* described from South America, Roland Thaxter collected a Chilean truffle in 1906 that fits morphologically in the genus *Gymnohydnotrya* (Smith & Pfister, unpublished data). Thaxter’s specimen may correspond to the ectomycorrhizal symbiont sequenced from *Nothofagus obliqua* roots in Argentina or may point to further undescribed diversity in the/gymnohydnotrya lineage. Zhang and Minter [67] suggested that *Gymnohydnotrya* belonged within the *Helvellaceae* but also suggested possible affinities with *Hydnotrya* (*Discinaceae*). Our analysis indicates that *Gymnohydnotrya* species actually belong to the previously unknown Southern hemisphere lineage (/gymnohydnotrya). We estimate the initial divergence of the Southern hemisphere *Tuberaceae* (/labyrinthomyces) at 43 Mya and/gymnohydnotrya at 72 Mya, with radiations during the Paleogene. This would coincide with the radiation of the Southern hemisphere genus *Nothofagus* (40–55 Mya) and the fragmentation of South America, Australia, and Antarctica (30–50 Mya) [69].

## Summary

In this study we reassessed the biogeography and origin of the *Tuberaceae* and their relatives using multiple loci and a global sampling of taxa. Multiple independent transitions from an aboveground to a belowground truffle fruiting body form have occurred in the *Tuberaceae* and in its newly recognized sister lineage/gymnohydnotrya. Our data indicate that the *Tuberaceae* most likely radiated from a common angiosperm-associated ectomycorrhizal ancestor in the late Jurassic. Subsequent radiations of major clades within the family have occurred on different continents during the Cretaceous and Paleogene, periods when many ectomycorrhizal angiosperm groups were also radiating. Several long-distance and intercontinental dispersal events have since occurred in several of the major clades within the *Tuberaceae*, including/puberulum and/choiromyces. We hypothesize that, in some cases, dispersal events of ancestral truffle species may have been correlated with host plant migration (e.g. with the migration of *Salix* into the Southern hemisphere), but that in other cases host switching may have facilitated intercontinental diversification through founder events. Finally, we have identified an epigeous species belonging to the *Tuberaceae* (*Nothojafnea* cf *thaxterii*), providing the first evidence that the *Tuberaceae* is not composed strictly of truffle fungi.

## Supporting Information

Table S1 Collection information and GenBank accession information for taxa sampled.
(XLS)

Table S2 Primers used in this study.
(XLS)

Table S3 Economically important *Tuber* species determined by 2009 market prices (USD) in the USA.
(XLS)
